# Supplementary material for: Finding women in fishing communities around Lake Victoria: “Feasibility and acceptability of using phones and tracking devices”
Source: PLoS One. 2024 Jan 11;19(1):e0290634. doi: 10.1371/journal.pone.0290634 (PMC10783786; doi:10.1371/journal.pone.0290634)
Supplement: Attachment — Submitted filename: Comments - Finding women in fishing communities around lake victoria.docx [file pone.0290634.s001.docx]

**Finding women in fishing communities around Lake Victoria: A qualitative study on the use of phones and tracking devises.**

This is an interesting paper with potential to inform projects or studies enrolling highly mobile participants – to achieve both high retention and improve validity of the study findings and or impact of the projects. Although the paper provides useful information on experiences and perceptions including feasibility and acceptability to use phones and tracking devices in studies, the manuscript requires major improvement before its consideration for publication.

**Title:** The title is well written and provides information on the population and the study setting as well as the design employed. I would suggest improving the title to identify with the objective of the study “*Finding women in fishing communities around Lake Victoria: feasibility and acceptability of using phones and tracking devises*”.

**Abstract:** The abstract is well structured and concise. Can revise the first sentence in methods section to read – ‘A formative qualitative ~~methods~~ study…’. It is necessary to clarify how many participants were enrolled in this study – Is it 64, 60 or 48?

**Introduction:** Generally, the introduction section is focused to what the manuscript is communicating. Provides a background to why the study is being conducted including the set-out objectives.

**Methods:** This section requires clarity. There are various sections that one is left wondering what was done and how was it done. First, the design is confusing, there seems to have been enrolment and follow up of participants either through FGDs or in-depth interviews for four months asking information related to their wellbeing, movements and experiences using the devices. However, the authors mention that this was a cross-sectional study while this seem to have had data collected over time with possibilities of experiences changing over time. It might be helpful to describe the type of phone provided by the study to participants as this could likely have had an effect on the findings of the study.

*Data collection and management* – the reader may benefit understanding who the research team that conducted the interviews and FGD was, what was their qualification including experience in conducting qualitative interviews. The fact that notes were taken during the interview and expanded after the interview meant that the quotes being provided at not verbatim, and that needs to be clarified. In line 158-177 provides the number of participants enrolled (18 women, 6 from each country randomized to simple study phone; another similar group – here I assume 18 to use their own mobile phone or a mobile phone they have access to; 24 women were given a GPS device) all these gives a total sample of 60 participants. Who was engaged in FGD and how many FGD were conducted in total? Were the same questions asked in IDIs and FGDs? How were the 4 participants in each site selected for IDIs? How was data saturation determined, if it was?

*Table 1:* This table seems to provide a total of 48 participants in the study, different from what is described above. There is a word missing between who and GPS on the second row (category column) “women who GPS while in the community”

**Results:** The first sentence reads, “We enrolled 48 women (24 per country) – the expected number here would be 64 (24*3 countries). The thematic area (e) experiences of those who carried GPS and phone is vague. Are these experiences unique from what is grouped in a-d? Beyond what is summarized on the table, concerns about male involvement is provided as a theme/group.

*Table 2* – it might be useful to categorize experiences of those who used their own phones vs. those who used the study phone (may necessitate providing three columns with phone users broken down into two)

Line 259-262 provides a quote from Youn men FGD, Kenya. In the manuscript all the study participants were women, and not exactly sure how males were involved in this study. This is supported by a statement in line 347-349 that men complained of not being informed as they did not have time to attend meeting that were organized at the beginning of the study. All these procedures including inclusion of men in the study is missing in the methods section.

In various instances, the descriptions provided do not match the quotes that exemplifies what is being described. The description on fears, worries and uncertainties around the device – line 296-297 describes an anticipated worry. At what point is the participant providing this description? After receipt of the device or before? It is my understanding that the participant would narrate experiences using a phone or gadget for tracking. Were they being asked a ‘what if’ question to necessitate the response received? In other instances, for example line 339-340 provides a description that is non-related to concerns about male involvement as a theme.

Line 316 provides a quote from Young women in Uganda – were participants in this study grouped into young and old? What were the age categories? Provide more details in the methods to help with this understanding.

The quotes seem to be quoted verbatim…. Not exactly sure how these were captured in a scenario where study team was taking notes and later expanding the notes taken ---- amounting to paraphrasing the participant’s verbal words.

**Discussion:** This section is fairly done. It might benefit from better situating this current study within the available literature. There are studies being compared to the current study, without contextualizing these differences. For example line 373-375, states the difference from this study with one that was conducted among the patient/caregivers on the ‘shape’ that was seen to be ‘big’. It is unclear if the current study used the same/similar GPS device as the Alzheimer’s study

Line 386 mentions GPS device requiring study staff to pick them up for charging and data download. This information is not provided in the methods section. Line 398 provides information on comprehending text messages – were the participants sent messages? Were they meant to read and respond to them? In the methods, the authors mention calling participants on specific time intervals but not text messaging is mentioned to warrant this.

Line 404-406, discussing the importance of sensitizing men in women’s participation in research is too general and not informed by this study. This study focused on a device or provision of a phone, something that identified participants in the study among their male partners. This may not be true should a study require say, one-time information related to women without follow up information.

Strengths and limitations to this study is too vague. How was this study able to ‘evaluate’ the feasibility? Rewrite this section.

The last paragraph before acknowledgement I assume is the conclusion and should be titled as such. It reads ok although it needs copyediting in some areas …with preference of using their own phones if community is sensitized? Currently reads sensitization.
