# Supplementary material for: Finding women in fishing communities around Lake Victoria: “Feasibility and acceptability of using phones and tracking devices”
Source: PLoS One. 2024 Jan 11;19(1):e0290634. doi: 10.1371/journal.pone.0290634 (PMC10783786; doi:10.1371/journal.pone.0290634)
Supplement: Attachment — Submitted filename: Revised Response to Reviewers.docx [file pone.0290634.s002.docx]

**Dear Editor, Thank you very much for the useful comments on our paper. We have responded to each comment as indicated below. Where we include line numbers, we refer to the track change version of the paper. We hope that the reviewers find our revisions adequate.**

**Reviewer #1:** Congratulations on undertaking an innovative important study. Your paper, including the introduction, methods, findings and discussion are all methodologically sound, logical and clearly articulated. This paper was a pleasure to read and the findings will be highly applicable to future studies and interventions in your region. There were a few grammatical errors. I have made suggested corrections in the comments below that do not request a re-write by the authors.

**Response: Thank you**

Line 265: However, majority of the women liked the size of the GPS much as they made suggestions for consideration to further reduce to a size of a Subscriber Identity module (SIM) card or ring. This sentence does not make sense and needs to be re-written.

***Response: Thank you for the observation, I have re-written the section line 318-320. It reads “However, majority of the women suggested the need to reduce the GPS size to a size of a Subscriber Identity module (SIM) card or finger ring”***

Line 374: a study conducted among patient/caregivers where the patient focused

***Response: Thank you, the correction has been considered line 432***

Line 385: While both devices require charging at the individual level,

***Response: Thank you, the correction has been considered line 445***

Line 388: However, basing on one of the studies conducted in fishing communities that compared physical tracing and phone use, phone use does not provide room for leaving messages with other persons 23 (family, workmates, community members) to relay to the participant in cases of no contact and ensure confidentiality (14). NEEDS RE-WORDING.

***Response: Thank you for the suggestion, the sentence is has been written line448-452. It reads “However, basing on one of the studies conducted in fishing communities that compared physical tracing and phone use, phone use does provide room for leaving messages with other persons (family, workmates, and community members) to relay to the participant. This compromises confidentiality (14). However, this can be solved by the use of GPS technology”***

Line 396: lack of constant electricity for charging

***Response: Thank you, the correction has been considered line 457***

Line 412: Overall, the women in these fishing communities embraced the use of phones and GPS devices as a mobility tracking method. Their preference was for using their own phones if community sensitization had occurred and had a further recommendation of a change in colour and design of the GPS devices if it were to be used. Thus phones and GPS devices can used in tracking movement patterns of mobile populations.

**Response: Thank you. The statement has been revised line 478-484**

**Reviewer #2:** Line 34 -36 reads: “Sixty-four 35 women participated: 24 were given a study phone; 24 were asked to allow tracking. The following comments indicate why I think the manuscript is partly technically sound. The comments call for minor but very significant revisions

of their own 36 phones and 16 were provided with a portable GPS device to understand what is most preferred.” But Table 1 (line 181-182 ) reads 48 participants. What happened to the 16 participants? It would appear that there were design and ethics issues which could have been avoided with more robust community engagement in conceptualization and design of the study or/and by mainstreaming gender in this study. What was the justification for engaging women only in this study? In other words, why were men excluded from this study? Would this not imply that in the fishing communities, women are the spreaders of HIV? As the findings indicate, did the study not expose women to risk of domestic violence? And were the women who participated in this study HIV positive? Were they all married or having male sexual partners now that they indicate that their partners would object to their carrying devices and would question where they got money from to buy the devices

***Response: Thank you for the observation, the sample size has been corrected and the table has revised 189-201and line 228. It reads “ Basing on the suggestions from the community focus group discussions, the identified women were invited to participate in the study. One group of 24 women, 6 from each implementing research institute were randomized to simple study mobile phone provided by the research institute that included credit for telephone use and another similar group of 24 were asked to use their own mobile phones or a mobile phone they have access to and given roughly USD 1.5 worth of credit for telephone use. Each woman from the groups that received/or used their own phones was contacted by phone after three, six and nine days by the research team and asked about any movements made outside the community they live in over the past 3 days. In addition, face-to-face IDIs were conducted monthly. In addition, a total of 16 women, 4 from each implementing site in Kenya, Tanzania and Uganda were given a small portable GPS device to carry for 4 months (see Table 1) line 210. Women were instructed on how to hand the device and how often the research would collect the devices to download data and charge the GPS. Based on the choice made during community discussions, four women from each site were identified to be invited to take part in the study***

Why women only?

***Response: Thank you for the concern. studies conducted in fishing communities of Lake Victoria in East Africa have reported high HIV prevalence and incidence, with more than half of the women infected with HIV in some age groups reference are indicated ( 2 and 3) line 521-522. Many women in fishing communities are very mobile; they change places of work and sexual partners frequently, which results in increased risk of acquisition of HIV and other sexually transmitted infections as indicated in the introduction but viewed as the only spreaders.***

***The methods section design line 122-125 and Study setting 153-156 indicates how men were involved.***

What is the goal of the study? Sometimes it is stated as “to inform future research innovations and interventions.’ (line 308). Does this mean all kinds of research innovations and interventions or health research innovations and interventions or both health research and health care innovations and innovations? There is need for clarity on this. Mixing health care with health research is particularly dangerous because it could lead to therapeutic misconception. Moreover, would the results be the same if the study was on health care only or health research only or both? I would imagine so. Asking me to carry a device for purposes of improving research is different from asking me to carry a device for purposes of improving health care. In other areas, there is reference to clinical research and clinical trials and public health and yet in other areas the focus is limited to informing methods, to informing larger intervention studies, to informing design of future studies etc. It is important to set the bounds of this study clearly.

***Response: Thank you. The goal to inform future health research innovations. Revisions have been made on line 32, 168-169 and 172-173, 440 and 446***

The study design is not that of an assessment but of an exploratory cross sectional descriptive study. The study explores and describes the experiences and perceptions of women in use of phones and GPS to track mobility.

***Response: Thank you for this observation, this was a multi-site formative qualitative study line 116***

Line 156 suggests that training on ‘ethical issues that would be encountered during field work’ was conducted. It would not have been possible to know the ethical issues that would arise so training would only have been on ethical considerations. Ordinarily, no researcher is definite on the ethical issues that they are likely to find in the study.

***Response: Thank you for the guidance, this has been corrected line 186***

**Reviewer #3:** Finding women in fishing communities around Lake Victoria: A qualitative study on the use of phones and tracking devises**.**

This is an interesting paper with potential to inform projects or studies enrolling highly mobile participants – to achieve both high retention and improve validity of the study findings and or impact of the projects. Although the paper provides useful information on experiences and perceptions including feasibility and acceptability to use phones and tracking devices in studies, the manuscript requires major improvement before its consideration for publication.

**Title:** The title is well written and provides information on the population and the study setting as well as the design employed. I would suggest improving the title to identify with the objective of the study “*Finding women in fishing communities around Lake Victoria: feasibility and acceptability of using phones and tracking devises*”.

***Response: Thank you for the guidance. The tittle has been revised as suggested “Finding Women in Fishing Communities around Lake Victoria: “feasibility and acceptability of using phones and tracking devices”. See line 1-3***

**Abstract:** The abstract is well structured and concise. Can revise the first sentence in methods section to read – ‘A formative qualitative ~~methods~~ study…’. It is necessary to clarify how many participants were enrolled in this study – Is it 64, 60 or 48?

***Response: The first sentence has been revised, line 34. The total number of study participants who received the devices were 64 (48 participants in the phone arm and 16 the GPS arm). Changes have been made. See line 37***

**Introduction:** Generally, the introduction section is focused to what the manuscript is communicating. Provides a background to why the study is being conducted including the set-out objectives.

***Response: Thank you.***

**Methods:** This section requires clarity. There are various sections that one is left wondering what was done and how was it done. First, the design is confusing, there seems to have been enrolment and follow up of participants either through FGDs or in-depth interviews for four months asking information related to their wellbeing, movements and experiences using the devices. However, the authors mention that this was a cross-sectional study while this seem to have had data collected over time with possibilities of experiences changing over time. It might be helpful to describe the type of phone provided by the study to participants as this could likely have had an effect on the findings of the study.

***Responses: Thank you for this observation. This was a multisite formative qualitative study. We have made revisions on line 115. The phones were basic inexpensive mobile phones loaded with weekly credit of roughly 1.5 US dollars line 127-128***

*Data collection and management* – the reader may benefit understanding who the research team that conducted the interviews and FGD was, what was their qualification including experience in conducting qualitative interviews. The fact that notes were taken during the interview and expanded after the interview meant that the quotes being provided at not verbatim, and that needs to be clarified. In line 158-177 provides the number of participants enrolled (18 women, 6 from each country randomized to simple study phone; another similar group – here I assume 18 to use their own mobile phone or a mobile phone they have access to; 24 women were given a GPS device) all these gives a total sample of 60 participants. Who was engaged in FGD and how many FGD were conducted in total? Were the same questions asked in IDIs and FGDs? How were the 4 participants in each site selected for IDIs? How was data saturation determined, if it was?

***Response: We have included in the methods section under study setting line 139-145 that: This was a multi-site qualitative study conducted by investigators from the partners of the Lake Victoria Consortium for Health Research (LVCHR). LVCHR members include: i) Kenya Medical Research Institute (KEMRI); ii) Mwanza Intervention Trials Unit (MITU) in Tanzania; iii) International AIDS Vaccine Initiative unit within the Uganda Virus Institute (UVRI/IAVI); and iv) the Medical Research Council/Uganda Virus Research Institute Uganda Research Unit & London School of Hygiene and Tropical Medicine (MRC/UVRI & LSHTM) Uganda Research Unit.***

***We have still indicated under data collection on line 175-177 that; ‘The Consortia leadership conceived and designed the study. Each site had one senior social science/behavioral researcher who coordinated activities of the study. These comprised of protocol training, supervision of field teams in data collection and management activities.***

***There were four field researchers at each site, both male and female field researchers of various ages (ranging between 24 to 40), who were selected from the research teams at each of the participating institutions based on their experience and training. The field researchers had enough experience to conduct research in the study settings.’ We have included a flowchart (Fig 2) as an attachment indicating the structure of the research teams. Line 178-182***

Fig 3: Flowchart on structure of the research teams

Lake Victoria Consortium Leadership

Senior researcher

KEMRI

Senior researcher

UVRI/IAVI

Qualitative field

researchers (4)

Senior researcher

MITU

Senior researcher

MRC/UVRI &

LSHTM

Qualitative field

researchers (4)

Qualitative field

researchers (4)

Qualitative field

researchers (4)

***Besides, we have corrected the sample size line 186-199;* *One group of 24 women, 6 from each implementing research institute were randomized to simple study mobile phone provided by the research institute that included credit for telephone use and another similar group of 24 were asked to use their own mobile phones or a mobile phone they have access to and given roughly USD 1.5 worth of credit for telephone use. Each woman from the groups that received/or used their own phones was contacted by phone after three, six and nine days by the research team and asked about any movements made outside the community they live in over the past 3 days. In addition, face-to-face IDIs were conducted monthly. In addition, a total of 16 women, 4 from each implementing site in Kenya, Tanzania and Uganda were given a small portable GPS device to carry for 4 months (see Table 1) line 225. Women were instructed on how to hand the device and how often the research would collect the devices to download data and charge the GPS.***

***Based on the choice made during community discussions, four women from each site were identified to be invited to take part in the study.***

*Table 1:* This table seems to provide a total of 48 participants in the study, different from what is described above. There is a word missing between who and GPS on the second row (category column) “women who GPS while in the community”

***Response: The numbers in the table have been reconciled. The total number of participants who carried the devices were 64. See line 225. The missing word has been added***

**Results:** The first sentence reads, “We enrolled 48 women (24 per country) – the expected number here would be 64 (24*3 countries). The thematic area (e) experiences of those who carried GPS and phone is vague. Are these experiences unique from what is grouped in a-d? Beyond what is summarized on the table, concerns about male involvement is provided as a theme/group.

***Response: Thank you for this suggestion. The total number of study participants has been reconciled. We did however, change the numbers because we have 16 participants in four sites, so we enrolled 64 participants for both arms. See line 241***

*Table 2* – it might be useful to categorize experiences of those who used their own phones vs. those who used the study phone (may necessitate providing three columns with phone users broken down into two)

Line 259-262 provides a quote from Young men FGD, Kenya. In the manuscript all the study participants were women, and not exactly sure how males were involved in this study. This is supported by a statement in line 347-349 that men complained of not being informed as they did not have time to attend meeting that were organized at the beginning of the study. All these procedures including inclusion of men in the study is missing in the methods section.

***Response: The table has been broken into two for the column of phone. We have included a statement on how males were involved in the study under study setting line 152-154“. Although our study population comprised of women residents and /or working in fishing communities.***

***Men were also involved during entry meetings and Focus group community discussions where shared their views about the feasibility of using GPS and phone devices, identifying and how to approach women to participate in the study. A statement has been included in the methods section-sampling procedure line 157-158***

In various instances, the descriptions provided do not match the quotes that exemplifies what is being described. The description on fears, worries and uncertainties around the device – line 296-297 describes an anticipated worry. At what point is the participant providing this description? After receipt of the device or before? It is my understanding that the participant would narrate experiences using a phone or gadget for tracking. Were they being asked a ‘what if’ question to necessitate the response received? In other instances, for example line 339-340 provides a description that is non-related to concerns about male involvement as a theme.

***Response: This was before they carried the devices. In response to description, this has been revised under results line 327-331 “Other felt that their sexual partners would question why they are carrying such devices. Some were scared of losing the GPS. On the other hand, the phone users in both arms presented issues of inconsistent power supply, network challenges. Those who were provided with institutional phones expressed the burden of carrying two phones.***

Line 316 provides a quote from Young women in Uganda – were participants in this study grouped into young and old? What were the age categories? Provide more details in the methods to help with this understanding.

***Response: Thank for this observation, Yes, those who participated in the community FGDs were grouped according to young women, elder women, young men, elder men. See line 121-124 “*** ***The discussions were held with residents of FCs (young women and men 18-25 years of age, older women, and older men 26 years+) separately to explore acceptability of using GPS devices and phones and how best individuals could be identified and approached to participate in carrying the devices”.***

The quotes seem to be quoted verbatim…. Not exactly sure how these were captured in a scenario where study team was taking notes and later expanding the notes taken ---- amounting to paraphrasing the participant’s verbal words.

***Response: A statement on how voices were documented as quotes is included line 230-231under data management and analysis “An effort was made to maintain participants’ voices as quotes on interesting information that came up during the interview”***

**Discussion:** This section is fairly done. It might benefit from better situating this current study within the available literature. There are studies being compared to the current study, without contextualizing these differences. For example line 373-375, states the difference from this study with one that was conducted among the patient/caregivers on the ‘shape’ that was seen to be ‘big’. It is unclear if the current study used the same/similar GPS device as the Alzheimer’s study

**Response: The sentence has been revised line 428-432 “*However, our findings differed from a study conducted on acceptability of GPS technology among patient/caregiver where the patient complained of the device's shape which they found too big (26). The GPS size was more like a portable phone whereas ours was very small and easy to carry that study participants liked*”**

Line 386 mentions GPS device requiring study staff to pick them up for charging and data download. This information is not provided in the methods section. Line 398 provides information on comprehending text messages – were the participants sent messages? Were they meant to read and respond to them? In the methods, the authors mention calling participants on specific time intervals but not text messaging is mentioned to warrant this.

***Response: Thank you for this observation, a statement has been included under data collection and management line 208-209 “Women were instructed on how to hand the device and how often the research would collect the devices on a monthly to download data and charge the GPS. Women were met in a specified place on a monthly basis for 4 months and asked about their wellbeing, movements within the preceding 30 days and about their experience with carrying the device”***

Line 404-406, discussing the importance of sensitizing men in women’s participation in research is too general and not informed by this study. This study focused on a device or provision of a phone, something that identified participants in the study among their male partners. This may not be true should a study require say, one-time information related to women without follow up information.

***Response: Thank you, however, during the FGD, women clearly pointed out that it is important to sensitize men (sexual partners). Basing on our experience, it is a general issue and relevant that may require attention for future studies.***

Strengths and limitations to this study is too vague. How was this study able to ‘evaluate’ the feasibility? Rewrite this section.

***Response: Thank you, this has been revised line 462-463. It reads “This study was able to determine the feasibility and acceptability of using phones and tracking devices for tracking participants, in a HIV high-risk population that are potentially eligible for future clinical trials”***

***Added a limitation line 467-470 which states “However, one of the limitations of this study we spotted was, while we gathered data from a number of sites along Lake Victoria, the study could not allow for direct comparison between sites to establish if there is noticeable differences in women’s perspectives on GPS and phone technology”***

The last paragraph before acknowledgement I assume is the conclusion and should be titled as such. It reads ok although it needs copyediting in some areas …with preference of using their own phones if community is sensitized? Currently reads sensitization.

***Response: Much appreciated for your observation, the sentence has been revised line 468-480. It reads “Overall, the women in these fishing communities embraced the use of phones and GPS devices as a mobility tracking method and preferred using their own phones. However, they recommended that the community members be sensitized to avoid misunderstandings. Participants may be concerned that their partners or other associates will not understand the research goals so providing community wide information can help to dispel concerns. It was also recommended to change the colour and design of the GPS devices if it were to be used in future, to make them less obvious and avoid question about what they were. Thus phones and GPS devices can used in tracking movement patterns of mobile populations. ”***
