# Supplementary material for: Finding women in fishing communities around Lake Victoria: “Feasibility and acceptability of using phones and tracking devices”
Source: PLoS One. 2024 Jan 11;19(1):e0290634. doi: 10.1371/journal.pone.0290634 (PMC10783786; doi:10.1371/journal.pone.0290634)
Supplement: Attachment — Submitted filename: Response to reviewers 20Jul2023.docx [file pone.0290634.s005.docx]

July 20th, 2023

Dear Academic Editor/Reviewer PLOS ONE,

**RE: Response to reviewers’ comments for manuscript number: PONE-D-22-04440R1**

We appreciate the comments and grateful to both the Editor and Reviewers who took the time to review our manuscript and provided supportive comments to improve the manuscript. The comments have been addressed point by point as indicated below. Where line numbers are included, we refer to the track change version of the paper. We hope that the reviewers find our revisions adequate.

Reviewers Comments:

Include Matt A Price: Thank you for the observation, Matt A Price has been included

Include Visual table: Visual table have been included line 291

Please include a legend for figure 3.

Thank you, this is already described in the manuscript. In the track copy, it’s indicated on line 175 to181

Reviewer #3: All comments are now addressed to my satisfaction. Would recommend including Table 2 provided in the response within the paper itself and referenced as such

**Response: Thank you so much for the recommendation, table 3 has been included in the paper. See line numbers 266-298 the table below has been included**

| **Table 3: Participant Characteristics** | | | | | | | | | |
| --- | --- | --- | --- | --- | --- | --- | --- | --- | --- |
| **Variables** | **MRC/UVRI and LSHTM** | | **UVRI-IAVI** | | **MITU** | | **KEMRI** | | **Total** |
|  | **Phone users** | **GPS users** | **Phone users** | **GPS users** | **Phone users** | **GPS Users** | **Phone users** | **GPS users** | **N = 64** |
| **Sex** |  |  |  |  |  |  |  |  |  |
| Male | 0 | 0 |  | 0 |  | 0 |  | 0 | 0 |
| Female | 12 | 4 | 12 | 4 | 12 | 4 | 12 | 4 | 64 |
|  |  |  |  |  |  |  |  |  |  |
| **Age (years)** |  |  |  |  |  |  |  |  |  |
| 18–24 | 5 | 2 | 3 | 1 | 2 | 2 | 2 | 1 | 18 |
| 25–34 | 3 | 1 | 5 | 2 | 6 | 1 | 5 | 2 | 25 |
| 35+ | 4 | 1 | 4 | 1 | 4 | 1 | 5 | 1 | 21 |
| **Education** |  |  |  |  |  |  |  |  |  |
| None | 1 | 0 | 0 | 0 | 0 | 0 | * | * | 1 |
| Primary | 5 | 3 | 8 | 2 | 6 | 2 | * | * | 26 |
| Secondary | 4 | 1 | 2 | 2 | 4 | 2 | * | * | 15 |
| Tertiary | 2 | 0 | 2 | 0 | 2 | 0 | * | * | 6 |
| **Occupation** |  |  |  |  |  |  |  |  |  |
| Fish related activities | 4 | 1 | 3 | 2 | 5 | 1 | 2 | 2 | 20 |
| Trader | 1 | 1 | 2 | 1 | 2 |  | 5 | 1 | 13 |
| Sex/bar worker | 4 | 2 | 5 | 1 | 3 | 2 | 2 | 1 | 20 |
| Other | 3 | 0 | 2 | 0 | 2 | 1 | 3 | 0 | 11 |
| **Marital status** |  |  |  |  |  |  |  |  |  |
| Married/cohabiting | 8 | 2 | 6 | 2 | 5 | 1 | 5 | 2 | 31 |
| Separated/single | 4 | 2 | 6 | 2 | 7 | 3 | 6 | 2 | 32 |
| Widowed | 0 | 0 | 0 | 0 | 0 | 0 | 1 | 0 | 1 |
|  |  |  |  |  |  |  |  |  |  |
| * Data not captured |  |  |  |  |  |  |  |  |  |
| FGD-Not collected |  |  |  |  |  |  |  |  |  |

Yours faithfully,

Gertrude Nanyonjo (on behalf of the co-authors)
