# Supplementary material for: Finding women in fishing communities around Lake Victoria: “Feasibility and acceptability of using phones and tracking devices”
Source: PLoS One. 2024 Jan 11;19(1):e0290634. doi: 10.1371/journal.pone.0290634 (PMC10783786; doi:10.1371/journal.pone.0290634)
Supplement: Attachment — Submitted filename: reviewer Comments.docx [file pone.0290634.s003.docx]

I still find the summary of the methods section in the abstract, main body methods section (line 112-122), sampling procedure (line 152-158) of the manuscript confusing due conflicting information provided.

Line 112-122 reads *“This study used community focus group discussions (FGDs) and in-depth interviews (IDIs) to document the perceptions, and experiences of women in fishing communities who carried either a GPS device or a phone. Community FGDs were considered appropriate for generating data on community perceptions regarding GPS devices and phones acceptability among women. The discussions were held with residents of FCs (young women and men 18-25 years of age, older women, and older men 26 years+) separately to explore acceptability of using GPS devices and phones and how best individuals could be identified and approached to participate in carrying the devices. IDIs were considered appropriate for generating data on personal lived experiences of those who actually carried the GPS devices, their own phones and study phones handed out by the researchers”*

Yellow highlight - I am left wondering how one would interview a male to document experiences of women who carried either phones or GPS devises?

The green highlight – Beyond this, I have not read anywhere who (participant characteristics), how they were selected, how many FGD conducted among them, what was asked of them among other information relevant to this group.

Your sampling procedure line 152-158 reads

*“The process of identifying participants started by conducting community entry meetings where we met the gatekeepers (both male and female) to obtain their support and buy-in. During community entry meetings, local leaders were requested to help in identifying women aged 18-45 years willing to carry GPS or phones and to help identify private and confidential meeting venues as well as mobilize potential participants. The study team screened the identified potential participants for uncoerced willingness to participate in the study as well as eligibility based on age above 18, at least 6 months residence in the community, own a phone and frequency of mobility.”*

In the highlighted section I get a sense that the gatekeepers were only approached to obtain support and buy in and that only women were recruited for the study and interviewed (IDI and or FGD) to explore their perceptions and experiences of using GPS devise and phone for tracking.

Your results section line 247-249 supports what I state above. Seems only women were recruited for this study or likely a sub-study of the main study.

*“We enrolled 64 women (16 per site) between 18-45 years of age in fishing communities of Kenya, Uganda and Tanzania and conducted community FGD with different groups of people to seek their views on the use of the two devices.”*

If this is a sub-analysis from a larger study, I would understand this confusion and there needs to be a delineation to every manuscript and its focus. If you are only focusing on women’s perception on use of the phones and GPS devices for tracking – including the pilot study to assess feasibility, then present this sub analysis only. As your male participants are not represented in the study population (64 participants), neither are they selected in the feasibility study, then remove their reference in the study.

The results section presenting the male participants in this study might be removed should the above be considered and similarly the discussion section referring to it.

Why was this study limited to direct comparison of data between sites? I believe the data was collected and segregated per site and as such if there were concerns raised by communities in one site compared to another, these could be shared. Otherwise, if these concerns cut across the three sites/countries – then can certainly say we did not find any differing views/perceptions/experiences of women in these communities hence the presentation of the results and discussion as such.
