# Supplementary material for: Finding women in fishing communities around Lake Victoria: “Feasibility and acceptability of using phones and tracking devices”
Source: PLoS One. 2024 Jan 11;19(1):e0290634. doi: 10.1371/journal.pone.0290634 (PMC10783786; doi:10.1371/journal.pone.0290634)
Supplement: Attachment — Submitted filename: Point_by_point_response to reviewer Comments 14June2023.docx [file pone.0290634.s004.docx]

June 14th, 2023

Dear Dr. Violet Naanyu, Academic Editor PLOS ONE

**RE: Response to reviewers’ comments for manuscript number: PONE-D-22-04440R1**

We appreciate the opportunity to respond to the comments and we are grateful to both the Editor and Reviewers who took the time to review our manuscript and provided helpful comments to improve the manuscript. All the comments have been addressed point by point as indicated below. Where we include line numbers, we refer to the track change version of the paper. We hope that the reviewers find our revisions adequate.

Editor(s)' Comments:

I still find the summary of the methods section in the abstract, main body methods section (line 112-122), sampling procedure (line 152-158) of the manuscript confusing due conflicting information provided.

**Response: Thank you for the observation. This has been revised see line 35-41**. **It reads “*Sixty-four (64) women participated in the study (16 per fishing community). Twenty-four (24) participants were given a study phone; 24 were asked to use their own phones and 16 were provided with a portable GPS device to understand what is most preferred. Women were interviewed about their experiences and recommendations on carrying GPS devices or phones. Twenty four (24) Focus Group Discussions with 8-12 participants were conducted, 4 FGDS in each fishing community with community members to generate data on community perceptions regarding GPS devices and phones acceptability among women. Data were analysed thematically and compared across sites/countries*”.**

Line 112-122 reads *“This study used community focus group discussions (FGDs) and in-depth interviews (IDIs) to document the perceptions, and experiences of women in fishing communities who carried either a GPS device or a phone. Community FGDs were considered appropriate for generating data on community perceptions regarding GPS devices and phones acceptability among women. The discussions were held with residents of FCs (young women and men 18-25 years of age, older women, and older men 26 years+) separately to explore acceptability of using GPS devices and phones and how best individuals could be identified and approached to participate in carrying the devices. IDIs were considered appropriate for generating data on personal lived experiences of those who actually carried the GPS devices, their own phones and study phones handed out by the researchers”*

Yellow highlight - I am left wondering how one would interview a male to document experiences of women who carried either phones or GPS devises?

**Response: The methods sections has been revised 114-123 reads “*****This study used in-depth interviews (IDIs) to document the perceptions, and experiences of women in fishing communities who carried either a GPS device or a phone. Besides, community FGDs were considered appropriate for*** ***generating data on community perceptions regarding GPS devices and phones acceptability among women. The discussions were held with residents of FCs (young women and men 18-25 years of age, older women, and older men 26 years+) separately to explore their views on the acceptability of using GPS devices and phones by women. During the discussions, participants were also asked to suggest how best individuals could be identified and approached to participate in carrying the devices*”**

The green highlight – Beyond this, I have not read anywhere who (participant characteristics), how they were selected, how many FGD conducted among them, what was asked of them among other information relevant to this group.

**Response: Very sorry, we had missed including the participant characteristics. This has been resolved. See Table 1; Participant characteristics**

***Concerning how participants were selected, the number of FGD and what was asked, this has been clearly stated line 118- 123 “Besides, community FGDs were considered appropriate for generating data on community perceptions regarding GPS devices and phones acceptability among women. The discussions were held with residents of FCs*** ***(young women and men 18-25 years of age, older women, and older men 26 years+) separately to*** ***explore their views on the acceptability of using GPS devices and phones by women. During the discussions, participants were also asked to suggest how best individuals could be identified and approached to participate in carrying the devices”.***

***Then 186-200 “******We organized and conducted to 6 introductory meetings with community gate keepers and the community members in each of the six fishing communities. Each meeting consisted of 8–15 members who had lived in the fishing communities for at least 1 year. Those who participated in the introductory sessions were nominated by local leaders in the communities. During community meeting, contact persons were identifies to support in mobilizing individuals to part in the FGDs and those to carry GPS device and phones. We conducted 4 Focus Group Discussions in each of the fishing community to explore views from different groups of people on the acceptability of using GPS devices and phones by women, how communities would think about GPS device and how to identify the women who are mobile to participate in carrying GPS device. Group discussion participants were identified in two ways: (1) selected by community local leaders and, (2) referrals from introductory meetings of people who were knowledgeable on the subject of discussion. The groups were divided into four categories based on gender and age: young women (18–25), older women (25+), young men (18–25) and older men (25+). Basing on the suggestions from the community meetings, and FGDs, the contact persons identified during community meetings supported the process of mobilizing women to carry GPS and phones*”.**

| **Table 2: Participant Characteristics** | | | | | | | | | |
| --- | --- | --- | --- | --- | --- | --- | --- | --- | --- |
| **Variables** | **MRC/UVRI and LSHTM** | | **UVRI-IAVI** | | **MITU** | | **KEMRI** | | **Total** |
|  | **Phone users** | **GPS users** | **Phone users** | **GPS users** | **Phone users** | **GPS Users** | **Phone users** | **GPS users** | **N = 64** |
| **Sex** |  |  |  |  |  |  |  |  |  |
| Male | 0 | 0 |  | 0 |  | 0 |  | 0 | 0 |
| Female | 12 | 4 | 12 | 4 | 12 | 4 | 12 | 4 | 64 |
|  |  |  |  |  |  |  |  |  |  |
| **Age (years)** |  |  |  |  |  |  |  |  |  |
| 18–24 | 5 | 2 | 3 | 1 | 2 | 2 | 2 | 1 | 18 |
| 25–34 | 3 | 1 | 5 | 2 | 6 | 1 | 5 | 2 | 25 |
| 35+ | 4 | 1 | 4 | 1 | 4 | 1 | 5 | 1 | 21 |
| **Education** |  |  |  |  |  |  |  |  |  |
| None | 1 | 0 | 0 | 0 | 0 | 0 | * | * | 1 |
| Primary | 5 | 3 | 8 | 2 | 6 | 2 | * | * | 26 |
| Secondary | 4 | 1 | 2 | 2 | 4 | 2 | * | * | 15 |
| Tertiary | 2 | 0 | 2 | 0 | 2 | 0 | * | * | 6 |
| **Occupation** |  |  |  |  |  |  |  |  |  |
| Fish related activities | 4 | 1 | 3 | 2 | 5 | 1 | 2 | 2 | 20 |
| Trader | 1 | 1 | 2 | 1 | 2 |  | 5 | 1 | 13 |
| Sex/bar worker | 4 | 2 | 5 | 1 | 3 | 2 | 2 | 1 | 20 |
| Other | 3 | 0 | 2 | 0 | 2 | 1 | 3 | 0 | 11 |
| **Marital status** |  |  |  |  |  |  |  |  |  |
| Married/cohabiting | 8 | 2 | 6 | 2 | 5 | 1 | 5 | 2 | 31 |
| Separated/single | 4 | 2 | 6 | 2 | 7 | 3 | 6 | 2 | 32 |
| Widowed | 0 | 0 | 0 | 0 | 0 | 0 | 1 | 0 | 1 |
|  |  |  |  |  |  |  |  |  |  |
| * Data not captured |  |  |  |  |  |  |  |  |  |
| FGD-Not collected |  |  |  |  |  |  |  |  |  |

Your sampling procedure line 152-158 reads

*“The process of identifying participants started by conducting community entry meetings where we met the gatekeepers (both male and female) to obtain their support and buy-in. During community entry meetings, local leaders were requested to help in identifying women aged 18-45 years willing to carry GPS or phones and to help identify private and confidential meeting venues as well as mobilize potential participants. The study team screened the identified potential participants for uncoerced willingness to participate in the study as well as eligibility based on age above 18, at least 6 months residence in the community, own a phone and frequency of mobility.”*

In the highlighted section I get a sense that the gatekeepers were only approached to obtain support and buy in and that only women were recruited for the study and interviewed (IDI and or FGD) to explore their perceptions and experiences of using GPS devise and phone for tracking.

Your results section line 247-249 supports what I state above. Seems only women were recruited for this study or likely a sub-study of the main study.

*“We enrolled 64 women (16 per site) between 18-45 years of age in fishing communities of Kenya, Uganda and Tanzania and conducted community FGD with different groups of people to seek their views on the use of the two devices.”*

If this is a sub-analysis from a larger study, I would understand this confusion and there needs to be a delineation to every manuscript and its focus. If you are only focusing on women’s perception on use of the phones and GPS devices for tracking – including the pilot study to assess feasibility, then present this sub analysis only. As your male participants are not represented in the study population (64 participants), neither are they selected in the feasibility study, then remove their reference in the study.

The results section presenting the male participants in this study might be removed should the above be considered and similarly the discussion section referring to it.

**Response:** ***Different methods were employed. We first introduced the study through community meetings, held FGDs with community leaders/gate keepers and community members and later conducted IDI with only women who carried GPS and phones but at the same time had FGDs with community leaders. This has been clarified under the methods section***

Why was this study limited to direct comparison of data between sites? I believe the data was collected and segregated per site and as such if there were concerns raised by communities in one site compared to another, these could be shared. Otherwise, if these concerns cut across the three sites/countries – then can certainly say we did not find any differing views/perceptions/experiences of women in these communities hence the presentation of the results and discussion as such.

**Response: *Thank you so much, true we did not find any differing views /perceptions/experiences in the different communities.***

Yours faithfully,

Gertrude Nanyonjo (on behalf of the co-authors)
